# Antinociceptive activity of methanolic extract of Muntingia calabura leaves: further elucidation of the possible mechanisms

**DOI:** 10.1186/1472-6882-14-63

**Published:** 2014-02-20

**Authors:** Zainul Amiruddin Zakaria, Mohd Hijaz Mohd Sani, Manraj Singh Cheema, Arifah Abdul Kader, Teh Lay Kek, Mohd Zaki Salleh

**Affiliations:** 1 Department of Biomedical Sciences, Faculty of Medicine and Health Sciences, Universiti Putra Malaysia, 43400 Serdang, Selangor, Malaysia; 2 Department of Veterinary Preclinical Sciences, Faculty of Veterinary Medicine, Universiti Putra Malaysia, 43400 Serdang, Selangor, Malaysia; 3 Integrative Pharmacogenomics Institute (iPROMISE), Level 7, FF3, Universiti Teknologi MARA, Puncak Alam Campus, 42300 Puncak Alam, Selangor, Malaysia

**Keywords:** Muntingia calabura, Elaecocarpaceae, Methanol extract, Antinociceptive activity, Mechanisms of action

## Abstract

**Background:**

*Muntingia calabura* (Elaecoparceae) is a medicinal plant traditionally used, particularly, by the Peruvian people to alleviate headache and cold, pain associated with gastric ulcers or to reduce the prostate gland swelling. Following the recent establishment of antinociceptive activity of *M. calabura* leaf, the present study was performed to further elucidate on the possible mechanisms of antinociception involved.

**Methods:**

The methanol extract of M. calabura (MEMC) was prepared in the doses of 100, 250 and 500 mg/kg. The role of bradykinin, protein kinase C, pottasium channels, and various opioid and non-opioid receptors in modulating the extract’s antinociceptive activity was determined using several antinociceptive assays. Results are presented as Mean *±* standard error of mean (SEM). The one-way ANOVA test with Dunnett's multiple comparison was used to analyze and compare the data, with *P <* 0*.*05 as the limit of significance.

**Results:**

The MEMC, at all doses, demonstrated a significant (p < 0.05) dose-dependent antinociceptive activity in both the bradykinin- and phorbol 12-myristate 13-acetate (PMA)-induced nociception. Pretreatment of the 500 mg/kg MEMC with 10 mg/kg glibenclamide (an ATP-sensitive K^+^ channel inhibitor), the antagonist of μ-, δ- and κ-opioid receptors (namely 10 mg/kg β-funaltrexamine, 1 mg/kg naltrindole and 1 mg/kg nor-binaltorphimine), and the non-opioid receptor antagonists (namely 3 mg/kg caffeine (a non-selective adenosinergic receptor antagonist), 0.15 mg/kg yohimbine (an α_2_-noradrenergic antagonist), and 1 mg/kg pindolol (a β-adrenoceptor antagonist)) significantly (p < 0.05) reversed the MEMC antinociception. However, 10 mg/kg atropine (a non-selective cholinergic receptor antagonist), 0.15 mg/kg prazosin (an α_1_-noradrenergic antagonist) and 20 mg/kg haloperidol (a non-selective dopaminergic antagonist) did not affect the extract's antinociception. The phytochemicals screening revealed the presence of saponins, flavonoids, tannins and triterpenes while the HPLC analysis showed the presence of flavonoid-based compounds.

**Conclusions:**

The antinociceptive activity of MEMC involved activation of the non-selective opioid (particularly the μ-, δ- and κ-opioid) and non-opioid (particularly adenosinergic, α_2_-noradrenergic, and β-adrenergic) receptors, modulation of the ATP-sensitive K^+^ channel, and inhibition of bradikinin and protein kinase C actions. The discrepancies in MEMC antinociception could be due to the presence of various phytochemicals.

## Background

Pain, as defined by the International Association for the Study of Pain (IASP), is “an unpleasant sensory or emotional experience associated with actual or potential tissue damage, or described in terms of such damage.” The definition empathizes pain as a complex multidimensional sensory-perceptual phenomenon that represents a unique subjective experience for each individual [1]. Pain is usually considered as a warning signal of actual or perceived tissue damage. In other word, pain which is produced by an external stimulus can therefore elicit reflex and conscious avoidance reaction to protect the body from potential harms. Nevertheless, pain can occur in the absence of tissue damage, even though the experience may be described as if the damage has occurred [2].

Receptors have their respective field or defined area from which they receive information. These nerve endings respond to noxious stimuli and transmit the information via afferent or sensory fibers to the CNS [3]. The dorsal horn is the gray matter in the posterior aspect of the spinal cord which is highly involved in pain integration, modification and relay. Pain impulses exit dorsal horn and ascend the spinal cord to the higher processing centers of the brain. The predominant pathways for pain conduction are the spinothalamic tract which synapse in the thalamus, and the spinoreticulothalamic tract which synapses in the reticular formation. The distinction in function of these two paths is not known [4]. Through pharmacological manipulation, it is possible to alter pain by decreasing transmission of pain signals to the brain or by increasing the inhibitory signal from the CNS [5].

Focusing on natural products as an alternative to many medications has been a major interest among scientists nowadays. Natural products as referred to Holt and Chandra [6] are herbs, herbal concoctions, dietary supplements, traditional Chinese medicines or alternative medicines. Natural product research is guided by ethnopharmacological knowledge and has brought substantial contributions to drug innovation by providing novel chemical structures and/or mechanism of actions [7]. *Muntingia calabura*, the sole species in the genus *Muntingia,* has been widely used as a traditional medication in the Southeast Asia and tropical America [8,9] to treat headaches and gastric ulcer, and as an emmenogogue, antidyspeptic, antispasmodic, diaphoretic, tranquillizer and tonic [8,10].

Scientifically, various medicinal properties have been reported, including anti-tumor [8,11], antibacterial [12,13], anti-inflammatory, antipyretic and antinociceptive [14,15], antiproliferative and antioxidant [16], antihypertensive [17] and antiulcer [18] activities. With regards to the antinociceptive mechanisms, several papers have reported on the involvement of various receptor systems (e.g. opioid, atropine, phenoxybenzamine, yohimbine, pindolol, haloperidol and bicuculline), L-arginine/nitric oxide/cyclic guanosine monophosphate pathway, vanilloid receptors and glutamatergic system in the modulation of antinociceptive activity of *M. calabura* extracts, namely the aqueous and chloroform extracts [19-22]. Moreover, it is suggested that the synergistic effect of the bioactive compounds, flavonoids, saponins, tannins and steroids, played an important role in the observed activities [16]. Recently, we have proved that the methanol extract of *M. calabura* (MEMC) leaves possesses good therapeutic effect in reducing nociceptive response [23] and further study by Mohd. Yusof et al. [24] leads to the isolation of 4 flavonoid-based antinociceptive-bearing bioactive compounds, of which one is a new compound called calaburone (8-hydroxy-6-methoxyflavone) and three were known compounds, namely 5-hydroxy-3,7,8-trimethoxyflavone, 3,7-dimethoxy-5-hydroflavone and 2’,4’-dihydroxy-3’-methoxychalcone. In this study, we further evaluate the possible mechanisms involved in the antinociceptive activity of the MEMC.

## Methods

### Plant collection

The leaves of *M. calabura*, collected from its natural habitat in Shah Alam, Selangor, Malaysia, were reidentified by Mr. Shamsul Khamis from the Institute of Bioscience (IBS), Universiti Putra Malaysia (UPM), Serdang, Selangor, Malaysia. A voucher specimen (SK 964/04) has been deposited in the Herbarium of the Laboratory of Natural Products, IBS, UPM, Serdang, Selangor, Malaysia.

### Preparation of plant extract

This procedure was carried out as described in detail by Zakaria et al. [19]. Briefly, 500 g of matured leaves that have been air-dried for 1-2 weeks at room temperature (27 ± 2°C) and grinded into powder were soaked in methanol in the ratio of 1:20 (w/v) for 72 hours. After that, the supernatant was filtered using steel filter, cotton, and Whatman no. 1 filter paper. The residue was subjected to the same procedures for another two times. The supernatant collected from each extraction was pooled together and then subjected to evaporation process using a rotary evaporator at 40°C under reduced pressure. The crude extract obtained was used to prepare the desired dose of treatment by dissolving them into 10% DMSO.

### Drugs and chemicals

The drugs apamin, charybdotoxin, tetraethylammonium chloride, atropine, haloperidol, pindolol, yohimbine, prazosin, phenylpherine, clonidine, caffeine, glibenclamide, β-funaltrexamine, naltrindole, nor-binaltorphimine, all purchased from Sigma Aldrich (U.S.A.) and bradykinin (Tocris Bioscience, U.K.), were prepared at the desired dose by dissolving them in distilled water (dH_2_O). Phorbol 12-myristate 13-acetate (Sigma Aldrich, USA) was dissolved in PBS solution. Acetic acid and dimethyl sulfoxide (DMSO) were purchased from Fisher Scientific (U.K.).

### Animals

Male ICR mice (25–30 g; 5–7 weeks old) and male Sprague-dawley rats (150-180 g) obtained from the Veterinary Animal Unit, Faculty of Veterinary Medicine, Universiti Putra Malaysia (UPM), Malaysia, and kept under room temperature (27 *±* 2°C; 70–80% humidity; 12 h light/darkness cycle) in the Animal Holding Unit (UPM), were supplied with food and water *ad libitum* up to the beginning of the experiments. The rats were, at all times, handled in accordance with current UPM guidelines for the care of laboratory animals and the ethical guidelines for investigations of experimental pain in conscious animals [25]. The study protocol of the present study was approved by the Animal House and Use Committee, Faculty of Medicine and Health Sciences, UPM (Ethical approval no.: UPM/FPSK/PADS/BR-UUH/00404). All experiments were conducted between 09.00 and 16.00 h to minimize the effects of environmental changes.

### Phytochemical and HPLC analysis of MEMC

#### Phytochemical screening of dried leaves and MEMC

The phytochemical screening of dried leaves of *M. Calabura* and MEMC was performed according to the standard screening tests and conventional protocols as adopted by Zakaria et al. [16].

### HPLC analysis of MEMC

The HPLC analysis of MEMC was performed according to method by Balan et al. [18] with slight modification. Briefly, 10 mg of MEMC was dissolved in 1 ml methanol and then filtered through the membrane filter (pore size 0.45 μm). A Waters Delta 600 with 600 Controller and Waters 2996 Photodiode Array (Milford, MA, USA) equipped with an autosampler, online degasser and column heater was used to analyze the filtered sample. Data was evaluated and processed using the installed Millenium 32 Software (Waters Product). The filtered samples were separated at 27°C on a minibore Phenomenex Luna 5 μm C_18_ column (dimensions 250 × 4.60 mm)\using a one-step linear gradient. The solvents were (A) 0.1% aqueous formic acid and (B) acetonitrile and the elution system was as follows: Initial conditions were 95% A and 5% B with a linear gradient reaching 25% B at t = 12 min. This was maintained for 8 min after which the programm decreased to 15% B at t = 22 min and was maintained for another 8 min. The programm then was returned to the initial solvent composition at t = 35 min. The flow rate used was 1.0 ml/min and the injection volume was 10 μl. The HPLC was monitored at 254 and 366 nm.

### Antinociceptive assays

#### Involvement of protein kinase C

The experiment was conducted based on the previously described method by Savegnago et al. [26]. A volume of 50 μl of PMA (a protein kinase C activator) solution (0.05 μg/paw) was injected into the ventral surface of the right hind paw of the rat 60 min after the oral administration of vehicle, ASA (100 mg/kg) or MEMC (100, 250 and 500 mg/kg). The animals were observed individually from 15-45 min following PMA injection and the amount of time the rat spent licking the injected paw was recorded using a chronometer.

### Bradykinin-induced nociception

Based on the method previously described by Ferreira et al. [27], bradykinin (10 nmol/paw in 50 μl) was injected into the plantar ventral surface of the right hind paw 60 min after the oral administration of vehicle, ASA (100 mg/kg) or MEMC (100, 250 and 500 mg/kg). The induced-rat was observed individually for 10 min, and the amount of time they spent licking the injected paw was recorded.

### Involvement of potassium channels

To determine the contribution of K^+^ channels in MEMC-induced antinociception a method previously described by Alves and Duarte [28] was used. Mice were pre-treated with glibenclamide (an ATP sensitive K^+^ channel inhibitor; 10 mg/kg, i.p.), apamin (an inhibitor of small conductance Ca^2+^-activated K^+^ channels, 0.04 mg/kg, i.p.), charybdotoxin (an inhibitor of large conductance Ca^2+^-activated K^+^ channels, 0.02, i.p.) or tetraethylammonium chloride (a non-selective voltage dependant K^+^ channel inhibitor, 4 mg/kg, i.p.) 15 min before oral administration of either vehicle or MEMC (500 mg/kg). Sixty minutes later, pain was induced using 0.6% acetic acid. The number of writhing was recorded for 25 min, 5 min following acetic acid injection.

### Effect of various receptor antagonists on MEMC-induced antinociception

The doses of drug administered to elucidate the possible involvement of the following receptor system was based on the method previously described by De Souza et al. [29], followed by pain induction using acetic acid-induced abdominal writhing test described by Mohd. Sani et al. [23]. Groups of animal were pre-treated with caffeine (3 mg/kg, i.p.), atropine (10 mg/kg, i.p.), haloperidol (20 mg/kg, i.p.), pindolol (1 mg/kg. i.p.), yohimbine (0.15 mg/kg, i.p), prazosin (0.15 mg/kg, i.p), phenylphrine (10 mg/kg, i.p.) or clonidine (0.15 mg/kg, i.p.) 15 minutes before the administration of MEMC (500 mg/kg, p.o). The pain was induced using 0.6% acetic acid 60 minutes after the administration of MEMC or vehicle. The number of writhing was counted cumulatively over the period of 25 minutes, 5 minutes following acetic acid injection.

### Analysis of opioid receptor subtypes

Evaluation of opioid receptor subtype involvement was done using an abdominal constriction test which is similar to previously described methods [30,31]. The doses of the opioid antagonists and timing of administration were based on previous studies conducted by Choi et al. [30] and Reeta et al. [31]. The μ opioid antagonist, β-funaltraxamine (β-FNA; 10 mg/kg, i.p.), δ opioid receptor antagonist, naltrindole (NALT; 1 mg/kg. i.p.) or κ opioid receptor antagonist, nor-binaltorphimine (nor-BNI; 1 mg/kg, i.p.) were administered 90 min, 15 min and 30 min respectively, before oral administration of 500 mg/kg of MEMC. The nociceptive stimuli was injected 60 minutes after MEMC administration.

### Statiscal analysis

The results are presented as Mean *±* standard error of mean (SEM). The one-way ANOVA test with Dunnett's multiple comparison was used to analyze and compare the data, with *P <* 0*.*05 as the limit of significance.

## Result

Phytochemical screening and HPLC analysis of MEMC

**Figure 1:**
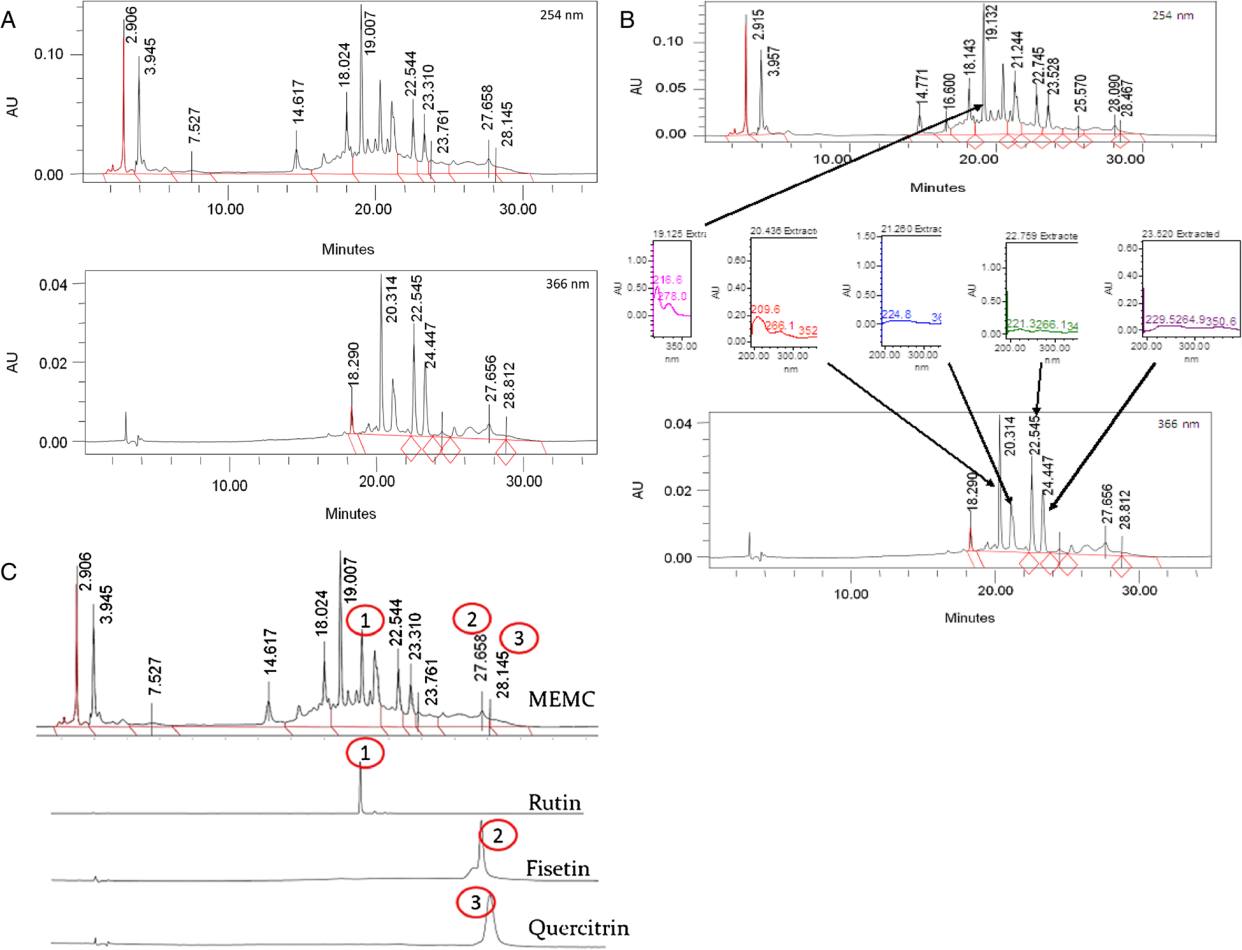
**The HPLC profile of MEMC. (A)**. The HPLC chromatograms of MEMC at the wavelengths of 254 and 366 nm.; **B)**. The UV spectra analysis of MEMC demonstrated the presence of five major peak, namely peak 1 (RT = 19.125 min), peak 2 (RT = 20.436 min), peak 3 (RT = 21.26 min), peak 4 (RT = 22.756 min) and peak 5 (RT = 23.52 min), which were observed at their respective λmax at the respective region of 216.6-278, 209.6-352.9, 224.8-364.2, 221.3-347 and 229.5-350.6 nm, suggesting, in part, the presence of flavonoid-based compounds.; and; **C)** Chromatogram of MEMC at 254 nm showing the presence of flavonoids type compounds, namely rutin, quercitrin, and fisetin, based on the comparison of their respective UV spectra analysis.

Table 1 showed the present of flavonoids, triterpenes, tannins, saponins and steroids in both the dried leaves and MEMC. There is, however, no alkaloids present based on the performed phytochemical screening. The HPLC profile of MEMC showed five major peaks at the wavelength of 254 and 366 nm (Figure 1A). The best isolation of the detected peaks (4 peaks) was observed at the wavelength of 366 nm. The four major peaks appeared in the chromatogram at the 366 nm wavelength tested at retention times of 20.436, 21.26, 22.756 and 23.52 min. Further analysis demonstrated that the five peaks showed λ_max_ values in the region of 216.6-278, 209.6-352.9, 224.8-364.2, 221.3-347 and 229.5-350.6 nm, respectively (Figure 1B).

**Table 1:** **Comparison on the phytochemical constituents between the leaves of *M. calabura *** **and MEMC**

**Phytochemical constituent**	**Sample**	**Result**	**Conclusion**
Alkaloid	MC	-	Not detected
	MEMC	-	Not detected
Saponin	MC	1+	Saponin was detected
	MEMC	1+	Saponin was detected
Flavonoid	MC	1+	Flavonoid was detected
	MEMC	1+	Flavonoid was detected
Tannins and polyphenolic compounds	MC	1+	Condensed tannins were detected
	MEMC	1+	Condensed tannins were detected
Triterpene	MC	3+	Triterpene was detected
	MEMC	1+	Triterpene was detected
Steroid	MC	3+	Steroid was detected
	MEMC	2+	Steroid was detected

For saponins: + – 1-2 cm froth; ++ – 2-3 cm froth; +++ – >3 cm froth.

For flavonoids, tannins, triterpenes and steroids: + – weak colour; ++ – mild colour; +++ – strong colour.

For akalioids: + – negligible amount of precipitate; ++ – weak precipitate; +++ – strong precipitate.

### Evaluation of protein kinase C and bradykinin receptor in MEMC-induced antinociception

**Figure 2:**
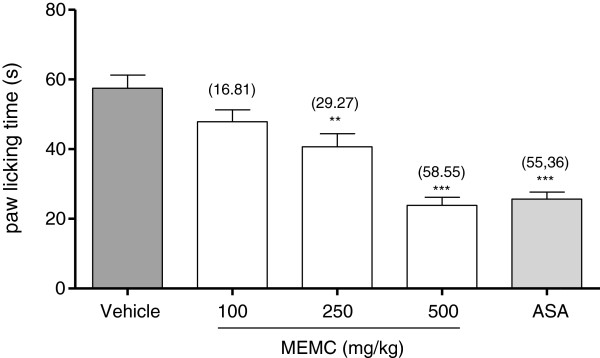
**The antinociceptive activity of MEMC against PMA-induced paw licking.** Each column represents the mean ± S.E.M. of six rats. Control (C: 10% DMSO, p.o.), Phorbol-12 myristate-13 acetate (PMA; 0.05 μg/50 μL/paw), MEMC (100, 250 and 500 mg/kg, p.o.) and acetylsalicylic acid (ASA: 100 mg/kg, p.o.). **, p < 0.01 and ***, p < 0.001 when compared to control group.

Figure 2 shows that the oral administration of 100, 250 and 500 mg/kg MEMC produced significant (p < 0.01 and p < 0.001) inhibition of PMA-induced nociception in rat. Interestingly, the 500 mg/kg MEMC exerted antinociceptive activity which was of similar intensity to that of 100 mg/kg ASA indicated by the similar percentage of analgesia recorded (58.55% and 55.36% respectively).

**Figure 3:**
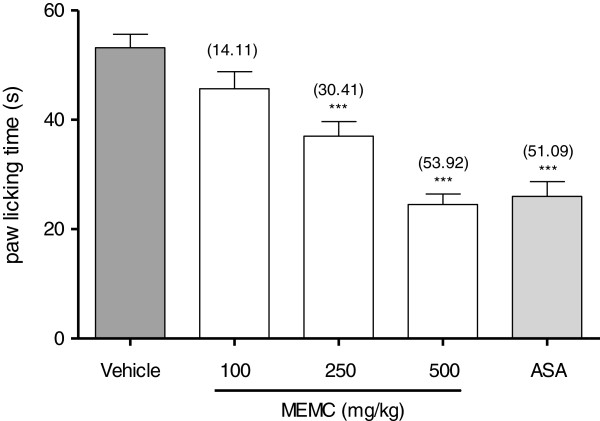
**The antinociceptive effect of MEMC against bradykinin-induced paw licking.** Each column represents the mean ± S.E.M. of six rats. Control (C: 10% DMSO, p.o.), bradykinin (10 nmol/paw), MEMC (100, 250 and 500 mg/kg, p.o.) and acetylsalicylic acid (ASA: 100 mg/kg, p.o.). ***, p < 0.001 when compared to control group.

As seen in Figure 3, MEMC given orally exhibits significant (p < 0.05) inhibition in a dose-dependant manner on the nociception caused by intra-plantar injection of bradykinin (10 nmol/paw) in rat. The maximal inhibition observed was 53.92% for the dose of 500 mg/kg MEMC. Similar inhibitory effect was observed for 100 mg/kg ASA.

### Involvement of potassium channels and, non-opioid and opioid receptors on MEMC-induced antinociception

**Figure 4:**
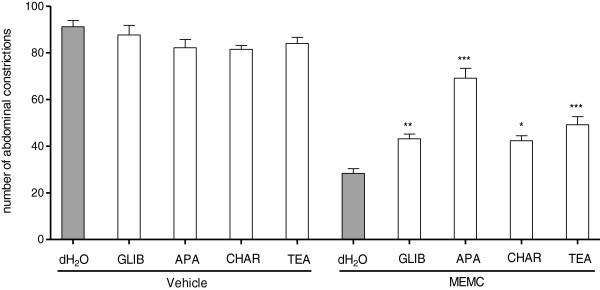
**The effect of pre-treatment with glibenclamide, apamin, charybdotoxin and tetraethylammonium chloride in MEMC-induced antinociception against acetic acid-induced abdominal writhing test in mice.** Each column represents the mean ± S.E.M. of six mice. Vehicle (10% DMSO, p.o.), MEMC (500 mg/kg, p.o.), glibenclamide (GLIB: 10 mg/kg, i.p.), apamin (APA: 0.04 mg/kg, i.p.), tetraethylammonium chloride (TEA: 0.01 mg/kg, i.p.). ***, P < 0.001, **, p < 0.01 and *, p < 0.05 when compared to MEMC-treated group.

Figure 4 shows the involvement of potassium channels in the modulation of MEMC-induced antinociceptive activity. Pretreatment with glibenclamide (10 mg/kg, i.p.), apamin (0.04 mg/kg, i.p.), charybdotoxin (0.02, i.p.) and tetraethylammonium chloride (4 mg/kg, i.p.), significantly (p < 0.01) reversed the antinociceptive activity of MEMC (500 mg/kg) when assessed using acetic acid-induced abdominal writhing test.

**Figure 5:**
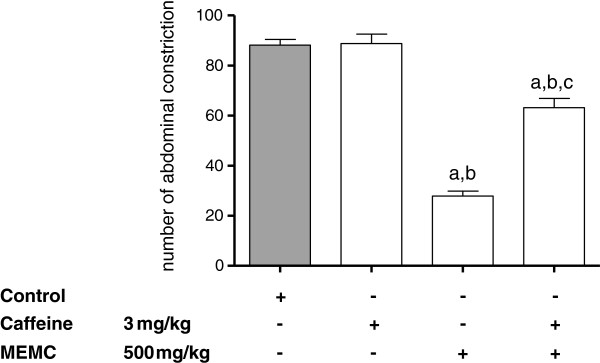
**The involvement of the adenosinergic system in MEMC-induced antinociception against acetic acid-induced abdominal writhing test in mice.** Each column represents the mean ± S.E.M. of six mice. Control (C: 10% DMSO, p.o.), Caffeine (3 mg/kg, i.p.), and MEMC (500 mg/kg, p.o.). ^a^p < 0.001 significantly different from control group; ^b^p < 0.001 significantly different when compared caffeine-treated group; ^c^p < 0.001 when compared to MEMC-treated group.

**Figure 6:**
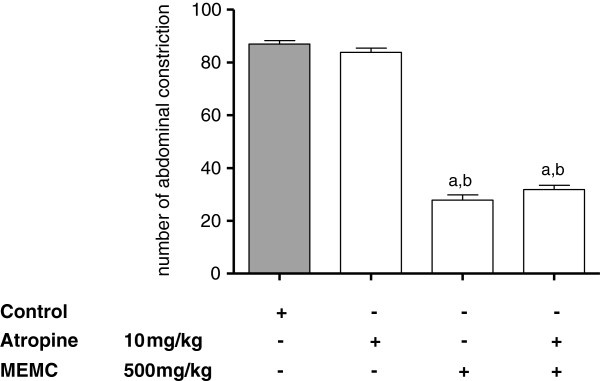
**The involvement of the cholinergic system in MEMC-induced antinociception against acetic acid-induced abdominal writhing test in mice.** Each column represents the mean ± S.E.M. of six mice. Control (C: 10% DMSO, p.o.), atropine (10 mg/kg, i.p.), and MEMC (500 mg/kg, p.o.). ^a^p < 0.001 significantly different from control group; ^b^p < 0.001 significantly different when compared atropine-treated group.

**Figure 7:**
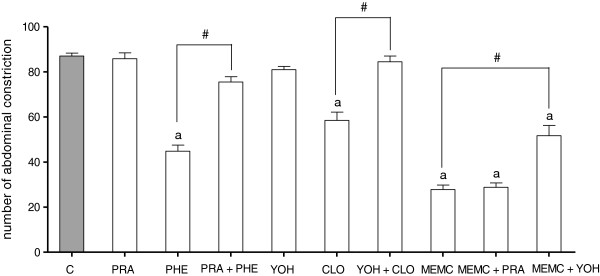
**The involvement of the adrenergic system in MEMC-induced antinociception against acetic acid-induced abdominal writhing test in mice.** Each column represents the mean ± S.E.M. of six mice. Control (C: 10% DMSO, p.o.), prazosin (PRA: 0.15 mg/kg, i.p.), Phenylphrine (PHE: 10 mg/kg, i.p.), Yohimbine (YOH: 0.15 mg/kg, i.p.), Clonidine (CLO: 0.15 mg/kg, i.p.) and MEMC (500 mg/kg, p.o.). ^a^p < 0.05 significantly different from control group; ^#^p < 0.05 significantly different when compared between respected treatment groups.

**Figure 8:**
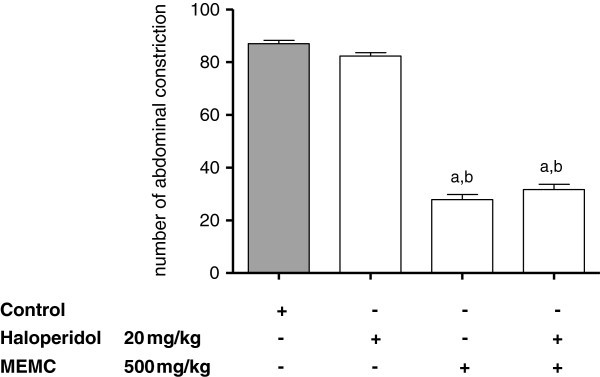
**The involvement of the dopaminergic system in MEMC-induced antinociception against acetic acid-induced abdominal writhing test in mice.** Each column represents the mean ± S.E.M. of six mice. Control (C: 10% DMSO, p.o.), Haloperidol (20 mg/kg, i.p.), and MEMC (500 mg/kg, p.o.). ^a^p < 0.001 significantly different from control group; ^b^p < 0.001 significantly different when compared haloperidol-treated group.

**Figure 9:**
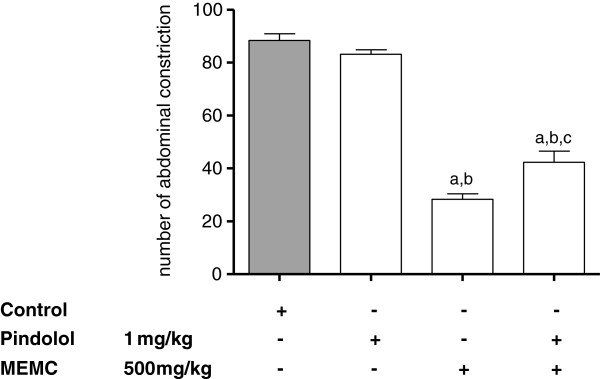
**The involvement of the serotonergic system in MEMC-induced antinociception against acetic acid-induced abdominal writhing test in mice.** Each column represents the mean ± S.E.M. of six mice. Control (C: 10% DMSO, p.o.), Pindolol (1 mg/kg, i.p.), and MEMC (500 mg/kg, p.o.). ^a^p < 0.001 significantly different from control group; ^b^p < 0.001 significantly different when compared pindolol-treated group; ^c^p < 0.01 when compared to MEMC-treated group.

**Figure 10:**
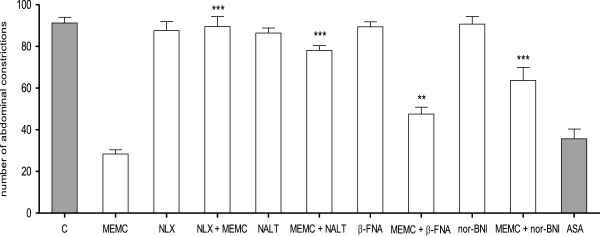
**Analysis of opioid receptor subtypes involvement in MEMC-induced antinociception against acetic acid-induced writhing test inn mice.** Control (C: 10% DMSO, p.o.), MEMC (500 mg/kg, p.o.), naloxone (NLX: 5 mg/kg, i.p.), naltrindole (NALT: 1 mg/kg, i.p.), β-funaltrexamine (β-FNA: 10 mg/kg, i.p.), nor-binaltorphimine (nor-BNI: 1 mg/kg, i.p.) and acetylsalicylic acid (ASA: 100 mg/kg, p.o.). ^***^P < 0.001 when compared to the group treated only with MEMC; ^**^p < 0.01 when compared to the group treated only with MEMC.

The antinociceptive activity of MEMC was also significantly (p < 0.05) reversed following intraperitoneal administration of 3 mg/kg caffeine (Figure 5), 10 mg/kg atropine (Figure 6), 0.15 mg/kg yohimbine (Figure 7), 0.2 mg/kg haloperidol (Figure 8), 0.2 mg/kg pindolol (Figure 9), or naltrindole, nor-binaltorphimine and β-funaltrexamine (Figure 10).

## Discussion

Previous studies conducted in our laboratory demonstrates the ability of the MEMC to produce significant antinociceptive activity in both chemicals- and thermal-induced nociception test model indicating possible participation of central and peripheral antinociceptive mechanisms. The present study focuses further on the mechanisms of action involved in antinociception induced by crude methanol extract from the leaves of *M. calabura*. The results obtained revealed that the oral administration of MEMC produced significant dose-dependant inhibition of intraplantar (i.pl.) injection of bradykinin- and PMA-induced nociception.

Protein kinase C (PKC) has been reported to indirectly involved in the central sensitization of normally silent N-methyl D-aspartate (NMDA) glutamate receptors located in the postsynaptic neuron [32,33], suggesting that the activation of PKC also play important role in the nociceptive transmission through a glutamatergic system. The i.pl. administration of PMA causes nociception, thermal hyperalgesia as well as mechanical allodynia in experimental animal model [34]. Based on the above statements, our data further strengthen the involvement of MEMC in central mechanism of antinociception as reported previously [23]. The activation of PKC occurs through interaction with intracellular lipid second messenger phosphatidylserine and diacylglycerol (DAG), and high level of calcium ions [35], which leads to the phosphorylation of many cellular components including the modulation of TRPV1 receptor [36-38]. Based on our findings, MEMC caused significant reduction in the nociceptive response induced by PMA in a dose-dependent manner, which in turn prevents the phosphorylation of TRPV1. This correlates well with our previous finding [23] where we demonstrated the possible involvement of TRPV1 in MEMC-induced antinociception through capsaicin-induced paw licking test. We, therefore, suggest that the MEMC antinociceptive activity involved partly the inhibition of TRPV1 receptor phosphorylation via attenuation of the PKC activation.

It is reported that PKC can also be directly activated by binding of bradykinin to its receptor [27]. This view was supported by our results demonstrating the MEMC's ability to suppress the nociception caused by bradykinin. Bradykinin is a potent inflammatory peptide messenger which is generated from a protein precursor, kallidin, through the action of specific enzyme kallikrein. During injury or inflammation, bradykinin will be released from the damaged tissues, from mast cells, as well as produced in the blood where it serves as vasodilators and increases vessel permeability [35]. This peptide is considered as one of the most potent pain-producing substance as it not only excites plenty of nociceptors, but also sensitizes them to other noxious stimuli through activation of B1 and B2 receptors [35,39].

Bradykinin acted through G-coupled protein receptor, on dorsal root ganglion (DRG) sensory neurons, elicits marked increase in Ca^2+^, through activation of DAG and PKC pathway [40,41]. Peripheral sensitization by bradykinin, which acted on the Aδ and C-fibers, evoke the release and synthesis of other second messengers, including prostaglandins, nitric oxide and neurokinins [40,42,43]. Pain induced through the introduction of bradykinin into the right hind paw of the experimental rat is significantly inhibited by oral administration of 250 and 500 mg/kg MEMC. It has been reported that the pain induced by bradykinin can be inhibited by cyclooxygenase (COX) inhibitor indomethacin [44], and therefore this type of pain is mediated by prostaglandins (probably PGE_2_). The ability of MEMC to inhibit bradykinin hyperalgesia correlates well with previous report [23], which proposed that MEMC antinociceptive activity seen in acetic acid-induced nociception may occurs through the inhibition of COX, as well as other mediators mentioned above or possibly by directly blocking the B_2_ receptors.

The noradrenergic receptor system involved greatly in descending modulation of pain pathways. Clonidine, a α_2_-adrenergic agonist, acting on the nerve endings of primary afferent fibers will inhibit the release of norepinephrine, glutamate and substance P, as well as pro-inflammatory cytokines resulting in sedative and analgesic actions [45,46]. Our findings suggested the involvement of α_2_-adrenergic, but excluded the α_1_-adrenergic receptors since MEMC activity was significantly reversed, when challenged with yohimbine (α_2_-adrenergic antagonist). In addition, serotonergic receptor pathway correlates with that of noradrenergic system. Activation of serotonergic receptor will cause the release of noradrenaline which activate postsynaptic α_2_-adrenergic in the spinal cord leading to antinociception [47,48], and pretreatment with pindolol (5-HT_1A/1B_ receptor/β-adrenoceptor inhibitor) significantly reversed MEMC antinociceptive activity indicating its role in serotonergic system.

We also demonstrated the involvement of adenosinergic receptor system in MEMC-induced antinociception. Caffeine, a non-selective adenosinergic receptor antagonist significantly reduced the action of MEMC. Pharmacologically, caffeine blocked adenosine A_1_, A_2A_, A_2B_, and A_3_ receptor but with lower affinity [49,50]. Adenosine receptor activation particularly A_1_ produces antinociception, by reducing PGE_2_[51,52] and triggers NO/cGMP/PKG/K_ATP_ pathway [53] in acute pain, and increases pain threshold [54] as well as inhibit glutamate release [55] in chronic pain. Adenosinergic and serotonergic systems are closely involved as A_1_ receptor antagonist can block serotonin analgesic action [56]. On the other hand, atropine (a cholinergic receptor antagonist) and haloperidol (a dopaminergic receptor antagonist) did not cause any significant changes in the number of abdominal constrictions, indicating lack of involvement of those receptor systems in MEMC antinociception.

The activation of 5-HT_1A_ has been shown to promote the opening of K^+^ channels and closing of Ca^+^ channels through coupling negatively to adenylyl cyclase which lead to sensory transmission inhibition [57]. Corroborating to the finding, we demonstrated that pre-treatment with glibenclamide (a specific ATP-sensitive K^+^ channel blocker), apamin (small conductance Ca^2+^-activated K^+^ channels), charybdotoxin (an inhibitor of large conductance Ca^2+^-activated K^+^ channels) and tetraethylammonium chloride (a non-selective voltage dependant K^+^ channel inhibitor) significantly reversed the antinociceptive effect of MEMC. The opening of ATP-sensitive K^+^ channel has been reported to participate in opioid-mediated antinociception, at the level of K^+^ and not opioid receptor [58], since specific ATP-sensitive K^+^ channel blockers (glibenclamide and gliquidone) shown to dose-dependently reduce the antinociceptive of morphine [59,60]. This correlates well with previous study demonstrating the involvement of MEMC in opioid receptor system [23].

Previously, we have demonstrated the involvement of opioid receptor in MEMC antinociception using non-selective opioid antagonist, naloxone [23]. In the present study, we elucidated the possible role of opioid receptor subtype in the modulation of MEMC antinociception using μ, δ, and κ opioid antagonists. Our findings demonstrated MEMC activity was significantly attenuated by all of the opioid subtypes' antagonists, suggesting the role of those receptors in the analgesic activity of MEMC. These receptors, found throughout the nervous system, spinal cord, midbrain and cortex, can mediate pain inhibition [61], and the report showed increased expression of δ opioid receptor when μ receptors were repeatedly activated [62], which accounted for the synergistic action seen in other studies [63,64].

The phytochemical screening shows the presence of flavonoids, triterpenes, saponins steroids and tannins which is in line with previous report [15], and interestingly all of these bioactive constituents has been reported to be involved in antinociceptive activity [65-68]. Our HPLC analysis revealed the possible presence of flavonols, namely rutin, quercitrin and fisetin. The ability of quercitrin to inhibit the pro-inflammatory mediators involved in pain modulation, especially cytokines, has been reported [69]. Rutin has been reported to produced antinociceptive activity by inhibiting both COX and lipooxygenase (LOX) pathways at high concentration [70]. Various reports have demonstrated that these types of flavonoids possess significant antinociceptive and/or anti-inflammatory activities [71-73].

## Conclusions

We conclude that the antinociceptive activity of MEMC may also be mediated through inhibition of PKC pathway and bradykinin receptor as well as through the activation of K^+^ channels, adrenergic, serotonergic and adenosinergic receptor systems. Our findings also revealed the possible interaction of MEMC with the three opioid receptor subtypes. The activity seen could be due to the synergistic effect of flavonoids, saponins, tannins and steroids.

## Competing interest

The authors declare that there is no competing interest.

## Authors’ contribution

MHMS carried out the experiments and drafted the manuscript. MSC, AAK, TLK and MZS participated in its design, involved in the statistical analysis and helped to draft the manuscript. ZAZ conceived of the study, participated in its design and helped to draft the manuscript. All authors read and approved the final manuscript.

## Pre-publication history

The pre-publication history for this paper can be accessed here:

http://www.biomedcentral.com/1472-6882/14/63/prepub
